# Challenges in Child Development Support and After-School Day Services Using Occupational Therapy: An Exploratory Analysis in Yamagata Prefecture, Japan

**DOI:** 10.7759/cureus.94103

**Published:** 2025-10-08

**Authors:** Saeko Takenaka, Tomoki Oda, Yasunobu Kase, Yumi Suzuki, Hiromi Fujii

**Affiliations:** 1 Occupational Therapy, Yamagata Prefectural University of Health Sciences, Yamagata, JPN; 2 Operations, Linie R, Inc., Tokyo, JPN; 3 Child Developmental Support, Linie R, Inc., Yamagata, JPN; 4 Child Developmental Support, Linie R, Inc., Kaminoyama, JPN

**Keywords:** child development support, community-based rehabilitation, family-centered support, icf, role of occupational therapists

## Abstract

Background

Japan’s Child Welfare Act ensures children’s rights to development and a nurturing environment. Child development support and after-school day services, established in 2012, provide developmental support for children. Occupational therapists (OTs), physical therapists (PTs), and speech-language pathologists (SLPs) utilize the International Classification of Functioning, Disability and Health framework to evaluate children’s activities and participation in their environment and to create effective support plans. However, there is a limited number of OTs, PTs, and SLPs available for these services. This study explored the challenges in child development support and after-school day services in Yamagata Prefecture, Japan, adopting an OT perspective.

Methods

Employing a mixed-methods approach, the researchers analyzed the support records of 222 children and their parents from two facilities. The study extracted data on children’s difficulties and parents’ characteristics and difficulties. Children’s difficulties were categorized into areas such as poor social communication and interpersonal relationship issues. Parental attitudes were classified as optimistic, diligent, negative, or indifferent, while parental difficulties included lack of knowledge and failure to recognize characteristics of their child’s disorder.

Results

The results indicated that interpersonal relationship issues were the most frequent challenge for the children, and lack of knowledge was the most common difficulty faced by parents. Notably, diligent parents showed a tendency to recognize their child’s characteristics but still had a lack of knowledge. Case studies highlighted the OT approach, focusing on both the child’s and parents’ needs, utilizing activities and environmental adjustments to improve the child’s functioning and parental understanding.

Discussion

This study highlights that children often face overlapping challenges, with self-injurious behavior reflecting complex factors. OTs and PTs address these through sensory and motor interventions, while parental characteristics strongly shape outcomes. Diligent parents, while recognizing their child’s needs, may lack access to necessary information. This suggests a need for improved access to tailored support services. This study underscores the importance of understanding the characteristics of both the child and the parent to provide effective support. Family support is crucial, alongside direct support for the child.

Conclusion

This study emphasizes the importance of family-centered support in child development support and after-school day services. It also notes the challenge of the shortage of OT and PT personnel in these settings. Future research should focus on multidisciplinary collaboration to create comprehensive support systems.

## Introduction

Echoing the spirit of the Convention on the Rights of the Child, Article 1 of Japan's Child Welfare Act guarantees that all children "have the right to be appropriately nurtured, to have their lives secured, to be loved and protected, and to have their healthy physical and mental growth and development, as well as their independence, and other welfare equally guaranteed." Adopting this fundamental framework, child development support (Jidō Hattatsu Shien) and after-school day services (Hōkago Day Service) were formally incorporated into the Act in April 2012 [1]. The former provides developmental support for preschool children in their communities, while the latter offers life enrichment and independence promotion for school-aged children with disabilities [2]. The stipulated roles of child development support, which include "guidance on basic daily living skills, provision of knowledge and skills, and training for adaptation to group life," must be "appropriate and effective ... according to the physical and mental condition of the child" [3]. Essentially, these services fulfill the Act's mandate by supporting the improvement of children's abilities after establishing a stable living foundation.

The main staffing in the child development support centers and after-school day services includes a child development support manager (CDSM), child care workers, and nursery teachers [4]. The role that the CDSM plays is to recognize the needs of children with disabilities and their parents they serve, and enable its staff to provide support based on the Child Development Support Plan. They also have the responsibility of managing the support process that is provided and conducting objective evaluations, etc. [5]. According to a survey by the Ministry of Health, Labor and Welfare, the proportion of holders of national caregiver qualification is the highest at 28.4%, followed by speech-language pathologists (SLPs) at 3.7%, occupational therapists (OTs) at 3.3%, and physical therapists (PTs) at 2.3% [6]. In other words, there are very few CDSMs who are OTs, PTs, or SLPs. In addition, this shows regional disparities [7]. It is inferred that this situation stems from underlying legal and systemic issues in Japan. In Japan, the limited number of OTs and PTs in child development support and after-school day services reflects systemic workforce distribution, where rehabilitation professionals are primarily allocated to hospital-based care rather than community services.

The authors have developed child development support centers and after-school day services led by OTs, PTs, and SLPs, primarily in Tokyo. However, in recognition of the regional disparities in child development support, the OT- and PT-centered child development support centers and after-school day services were opened in the cities of Kaminoyama in June 2019 and in Yamagata in April 2022. The number of child development support centers and after-school day services led by OTs, PTs, and SLPs within Yamagata Prefecture has remained limited to a few facilities. The advantage of OTs, PTs, and SLPs lies in their ability to apply the International Classification of Functioning, Disability and Health (ICF) to the creation of individual support plans. OTs, PTs, and SLPs can evaluate the activities and participation of target children, incorporating personal and environmental factors and utilizing this evaluation in the creation, implementation, and re-evaluation of support programs [8-10].

This study aims to clarify the characteristics of children and their parents targeted by child development support centers and after-school day services in Yamagata from an OT perspective and to explore the challenges in providing support within these services.

## Materials and methods

Research design

This study employed a mixed-methods approach to both grasping the overall trends of children’s difficulties (personal factors) and parents’ characteristics and difficulties (environmental factors) through quantitative data from an OT perspective, and understanding the content of support through qualitative data.

Participants

The participants in this study were initially 268 children and their parents who received support between June 2019 and October 2023 at two facilities for child development support and after-school day services operated by the Linie Group Corporation, based on the Child Welfare Act, in Yamagata Prefecture, Japan.

Of these, support records lacked sufficient data on children’s difficulties and/or parents’ characteristics and difficulties for 46 cases, which were therefore excluded. Consequently, the final analysis included 222 children and their parents.

To illustrate the interaction between children's difficulties and parental characteristics, two cases were selected from the 222 participants. The selection was conducted by OT, who classified cases according to the ICF domains, considering both personal factors (the child's difficulties) and environmental factors (parental characteristics and difficulties). These two cases, representing parents with comparable characteristics and a diligent approach, were chosen randomly because they exemplified frequently observed patterns in the dataset, thus allowing for an in-depth qualitative analysis aligned with the ICF framework.

Ethical considerations

This study was conducted with the approval of the Ethical Review Committee of Linie R, Inc. (Approval Number: 2087). All data obtained in the study were anonymized using numerical coding and symbolization to protect privacy and comply with ethical requirements. In addition, the data obtained were password-protected and kept on the researcher’s computer.

Analysis methods

Data Extraction Method

An OT first classified all participants using a systematic coding framework aligned with the ICF domains to ensure non-arbitrary case selection. An OT extracted information from users’ support records and categorized it into three domains: children’s difficulties, parents’ characteristics, and parents’ difficulties. The support records included the following: intake forms, interview records (records of basic information and needs of the child gathered before service provision), service utilization plans (master plans for the child’s support created by consultation support offices), individual support plans (support policies for the facility outlining the child’s assessment, support content, and goals), support records (records of the child’s condition and changes during service use), and monitoring sheets (records of reflections with parents after support implementation and the setting of subsequent goals). These records were standardized according to national guidelines for child development support services under the Child Welfare Act, which enhances consistency across cases. Support was provided based on these records, following a PDCA (Plan-Do-Check-Act) cycle.

The extracted data were analyzed retrospectively to examine the relationships between the characteristics and difficulties. Subsequently, we selected support cases taking into account parents’ characteristics and difficulties in relation to children’s difficulties. The effectiveness of these cases was analyzed qualitatively by examining the activities and participation within the framework of the ICF, analyzing the relationship between children’s difficulties and parents’ characteristics and difficulties.

Data Analysis Methods

Statistical analysis: Descriptive statistics were performed on basic attributes, such as the child’s age, gender, and presence of medical conditions. The number of target children was calculated for each child's difficulties, parents’ characteristics, and parents’ difficulties, categorized by the child’s age, gender, and medical condition. Gender was analyzed using the chi-squared test, and age was analyzed using the Kruskal-Wallis test. In addition, the number of overlapping items was calculated to understand the overall picture of each of the children’s difficulties and parents’ difficulties. Descriptive statistics were calculated for children’s age, gender, and presence of medical conditions, as well as for children’s difficulties, parents’ characteristics, and parents’ difficulties. Associations with gender were tested using the chi-squared test. Overlaps among children’s and parents’ difficulties were also assessed. All analyses were conducted using IBM SPSS Statistics for Windows, Version 29 (Released 2021; IBM Corp., Armonk, New York, United States), with the significance level set at 5%.

Overview of Each Domain

To understand the overall picture of the domains of children’s difficulties and parents’ difficulties, the number of overlapping items was calculated, and Venn diagrams were created.

## Results

Data extraction and classification

Children’s Difficulties

The extraction of children’s difficulties focused on activity-related challenges that were apparent in daily life. As a result, the extracted items were consolidated into five categories: difficulty with verbal communication (classified as poor social communication); interpersonal relationship issues stemming from the individual’s characteristics, leading to unsuccessful relationships (classified as interpersonal relationship); restlessness, strong temper tantrums (classified as restlessness or strong tantrums); being unable to find pleasure in interacting with others outside the family (classified as refusal to get involved outside of the family); and self-harm or harm to others is significant, making it consistently difficult to ensure the safety of the individual or those around them (classified as self-injurious behavior). In this domain, a single child could be classified under multiple heads.

Parents’ Characteristics

The extraction of parents’ characteristics focused on the parents' personality, as perceived by the OT, consolidated into four categories: optimistic, diligent, negative, and indifferent. Each parent was assigned to only one category.

Parents’ Difficulties

The extraction of parents’ difficulties was consolidated into six items: unwilling to acknowledge the child’s disability characteristics (classified as failure to recognize characteristics); lack of knowledge regarding engaging with a child with disabilities (classified as lack of knowledge); has own disability that makes understanding the child difficult classified as parent’s own disability, unable to adequately care for the child due to elderly care responsibilities (classified as issues in the home environment); child is a subject of the Council for Children Requiring Protection and support for the home environment is insufficient (classified as lack of support for family environment); and child is socially withdrawn and unable to attend the day service facility (classified as child withdrawal). Here, a single parent could be classified under multiple heads.

This information was integrated into a database, together with the basic attributes of the service users. Furthermore, the overall picture of children’s difficulties and parents’ difficulties, including overlapping items, was visualized in diagrams.

Data analysis

Statistical Analysis

The mean age ± standard deviation of the 222 participating children was 7.5 ± 3.0 years, with 176 boys (79%) and 46 girls (21%). Of these, 146 had a diagnosis, with autism spectrum disorder (ASD) being the most frequent, at 74 children, followed by attention-deficit/hyperactivity disorder (ADHD) and intellectual disability.

Data on children's difficulties (see Table 1) showed that interpersonal relationships were reported most frequently (182 children), followed by restlessness or strong tantrums (131 children) and poor social communication (73 children). Notably, restlessness or strong tantrums were significantly more common among boys (p=0.032).

**Table 1:** Basic attributes of children domains Note: An underscore (_) within an entry indicates a hierarchical relationship in the format "Category_Subcategory." For example, "Developmental Disorders_Autism" signifies "Autism" as a subcategory of "Developmental Disorders." ADHD: attention-deficit/hyperactivity disorder

Basic attributes		All member	Difficulties among children _Including duplicates				
			Interpersonal relationship	Restlessness or strong tantrums	Poor social communication	Refusal to get involved outside of the family	Self-injurious behavior
Number		222	182	131	73	71	31
Age	Mean±SD	7.5±3.0	7.7±3.0	7.1±2.5	6.6±3.1	7.5±2.9	7.7±2.9
Gender	Male	176	146	110	58	59	28
	Female	46	36	21	15	12	3
Presence or absence of children's diseases	declare_dignosis	146	129 (70.9%)	92 (70.2%)	49 (67.1%)	50 (70.4%)	25 (80.6%)
	declare_none diagnosis	21	17	13	4	4	5
	none declare	43	36	25	19	17	1
	none records	12	0	1	1	0	0
Diseases _Including duplicates	Intellectual Disability	40	33	26	21	17	11
	Developmental Disorders_Autism	4	4	4	4	1	4
	Developmental Disorders_Asperger	2	2	2	1	1	0
	Developmental Disorders_ADHD	50	48	17	9	14	11
	Developmental Disorders_ASD	74	63	49	26	28	12
	Developmental Disorders_Others	1	1	1	0	0	0
	Physical Disability	2	1	1	1	0	0
	Severe Mental and Physical Disabilities	0	0	0	0	0	0
	Others	38	35	20	15	12	3
	None	72	49	35	23	21	3
Therapists’ impressions of the parents	Diligent	129	109	76	49	48	18
	Negative	31	29	22	11	10	4
	Optimistic	26	26	20	6	4	3
	Indifferent	19	17	12	7	8	5
	None	14	1	1	0	1	1
Difficulties of the parents _Including duplicates	Lack of knowledge	155	133	100	58	49	20
	Failure to recognize characteristics	101	95	70	33	36	10
	Parent’s own disability	29	27	24	11	7	7
	Lack of support for family environment	11	11	9	4	2	3
	Child withdrawal	10	9	3	4	6	3
	Issues in the home environment because of elderly care	2	2	2	2	0	0

Among the parents’ difficulties (see Table 2), the most frequent item was a lack of knowledge (155 parents). This was followed by a failure to recognize (101 parents) and parents' own disability (29 parents). Notably, some parents’ difficulties were due to environmental factors, such as family problems arising from caregiving, rather than any issue directly related to the parents themselves.

**Table 2:** Basic attributes of parent's domains_difficulties of the parents Note: An underscore (_) within an entry indicates a hierarchical relationship in the format "Category_Subcategory." For example, "Developmental Disorders_Autism" signifies "Autism" as a subcategory of "Developmental Disorders." ADHD: attention-deficit/hyperactivity disorder

Basic attributes		Difficulties of the parents _Including duplicates					
		Lack of knowledge	Failure to recognize characteristics	Parent’s own disability	Lack of support for family environment	Child withdrawal	Issues in the home environment because of elderly care
Number		155	101	29	11	10	2
Gender of children	Male	123	79	17	9	8	2
	Female	32	22	12	2	2	0
Presence or absence of children's diseases	declare_dignosis	105 (67.7%)	73 (72.3%)	21 (72.4%)	7 (63.6%)	8 (80.0%)	0
	declare_none diagnosis	14	11	2	2	0	0
	none declare	34	17	6	2	2	2
	none records	2	0	0	0	0	0
Children's diseases _Including duplicates	Intellectual Disability	29	20	2	2	0	0
	Developmental Disorders_Autism	2	2	0	0	0	0
	Developmental Disorders_Asperger	2	1	0	0	0	0
	Developmental Disorders_ADHD	38	21	5	3	3	0
	Developmental Disorders_ASD	49	41	14	6	7	0
	Developmental Disorders_Others	1	1	0	0	0	0
	Physical Disability	0	1	0	0	0	0
	Severe Mental and Physical Disabilities	0	0	0	0	0	0
	Others	29	19	7	3	3	0
	None	47	26	7	3	2	2
Therapists’ impressions of the parents	Diligent	99	49	11	7	10	0
	Negative	20	23	9	2	0	0
	Optimistic	21	19	3	0	0	0
	Indifferent	14	10	6	2	0	2
	None	1	0	0	0	0	0

Regarding parents’ characteristics (see Table 3), diligence was the most frequent, accounting for 58%. This was followed by negative, optimistic, and indifferent, in descending order.

**Table 3:** Basic attributes of parent's domains_therapists’ impressions of the parents Note: An underscore (_) within an entry indicates a hierarchical relationship in the format "Category_Subcategory." For example, "Developmental Disorders_Autism" signifies "Autism" as a subcategory of "Developmental Disorders." ADHD: attention-deficit/hyperactivity disorder

Basic attributes		Therapists’ impressions of the parents				
		Diligent	Negative	Optimistic	Indifferent	None
Number		129	31	26	19	14
Gender of children	Male	108	23	17	14	12
	Female	21	8	9	5	2
Presence or absence of children's diseases	declare_dignosis	91 (70.5%)	25 (80.6%)	15 (57.7%)	10 (52.6%)	5 (35.7%)
	declare_none diagnosis	11	2	5	2	1
	none declare	26	4	6	7	0
	none records	1	0	0	0	8
Children's diseases _Including duplicates	Intellectual Disability	25	5	3	5	3
	Developmental Disorders_Autism	4	0	0	0	0
	Developmental Disorders_Asperger	2	0	0	0	0
	Developmental Disorders_ADHD	29	7	8	5	1
	Developmental Disorders_ASD	41	15	4	4	2
	Developmental Disorders_Others	1	0	0	0	0
	Physical Disability	1	0	0	0	0
	Severe Mental and Physical Disabilities	0	0	0	0	0
	Others	23	6	3	1	0
	None	35	6	10	9	9

Overview of the Domains

**Figure 1:**
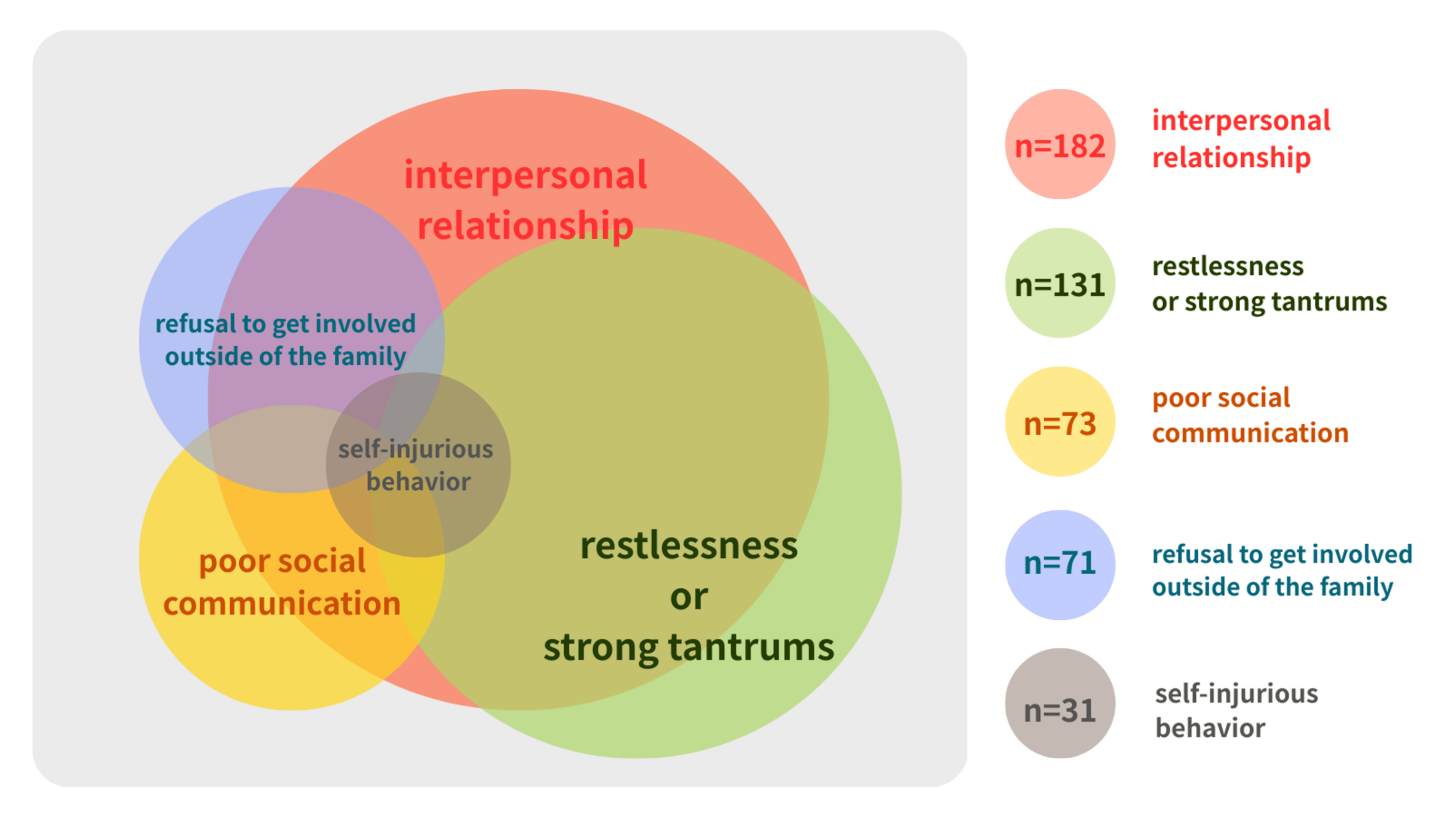
Relationships among items for difficulties among children

**Figure 2:**
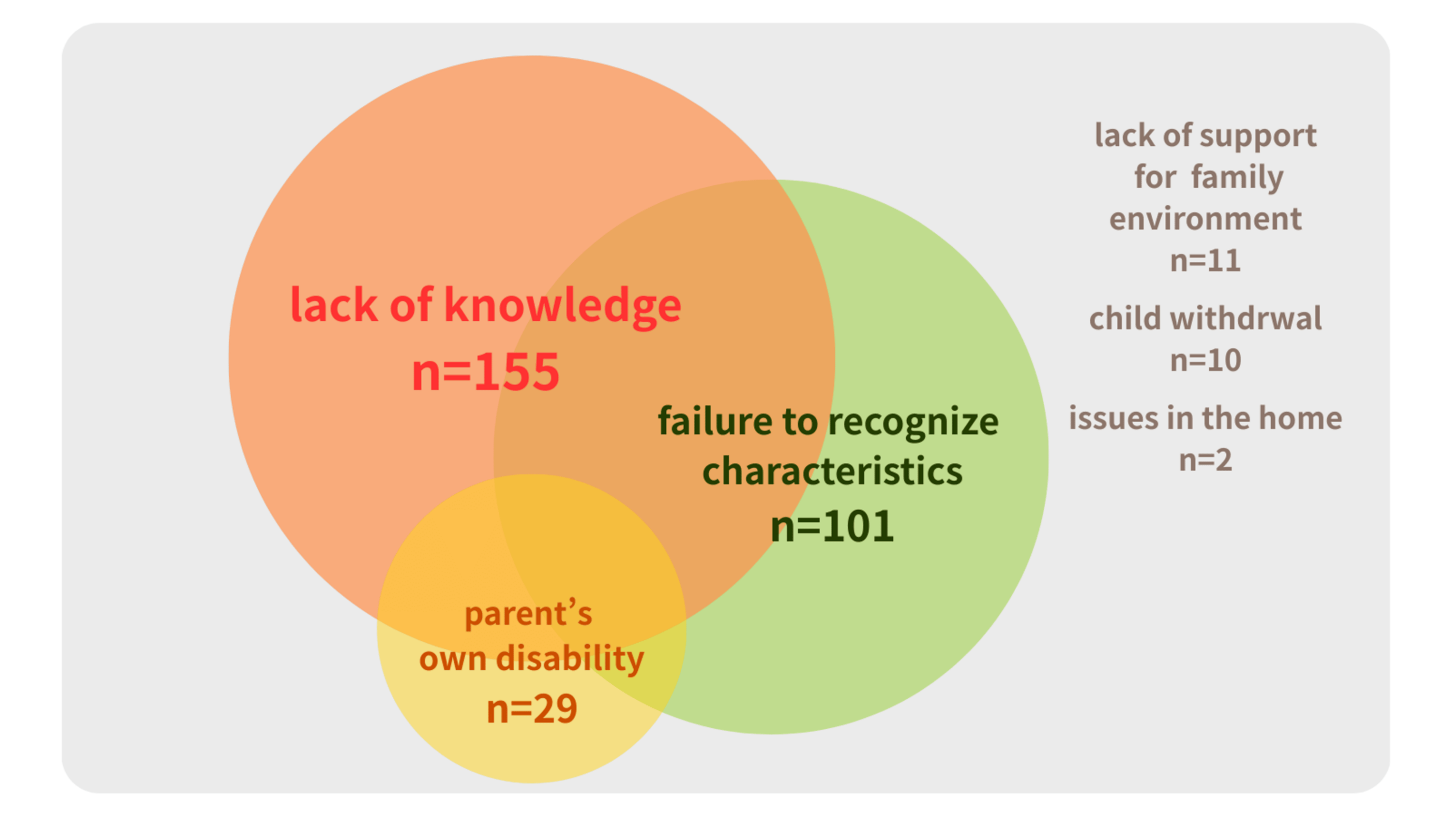
Relationships among items for parental difficulties for all

There were overlapping items seen within the domains of children’s difficulties and parents’ difficulties. As shown in Figure 1, it became clear that among the children's difficulties, self-injurious behavior overlapped with all other difficulties (poor social communication, interpersonal problems, restlessness or severe tantrums, refusal to interact with people outside the family). This suggests that self-injurious behavior arises from a combination of multiple complex factors. Similarly, multiple items overlapped in parental difficulties (Figure 2), with particularly strong mutual associations observed between lack of knowledge, difficulty recognizing characteristics, and parental disability.

Relationship between parents’ characteristics and parents’ difficulties

**Figure 3:**
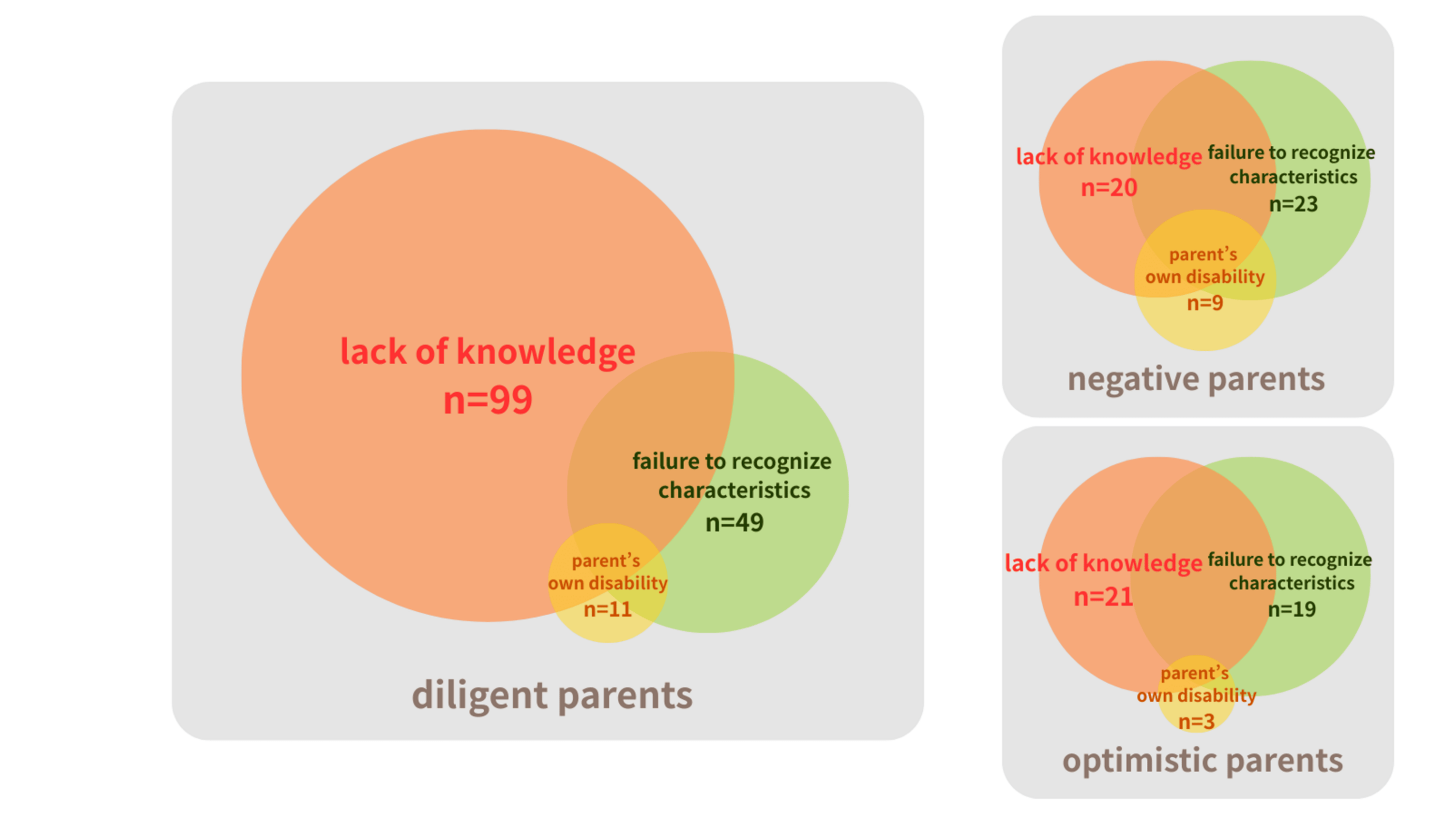
Associations among items for parental difficulties

Analysis of the relationship between parental characteristics and difficulties revealed that, as shown in Table 4, over 50% of optimistic, negative, and indifferent parents reported multiple difficulties. In contrast, among diligent parents, only lack of knowledge was reported at a frequency exceeding 50% (76.7%). Furthermore, the visualization in Figure 3 indicates that diligent parents tend to have less overlap between knowledge gaps and difficulties in recognizing characteristics compared to optimistic and negative parents. This strongly suggests that diligent parents recognize their child's characteristics but may lack access to appropriate support information.

**Table 4:** Associations of parent's difficulties with therapists’ impressions of the parents The bold values indicate the percentages in the “Yes” column that exceeded 50% for parents within the "Diligent," "Optimistic," "Negative," and "Indifferent" characteristic groups.

Therapists’ impressions of the parents' categories	Difficulties among parents' categories		Frequency (n)	Percentage (%)
Diligent	Failure to recognize characteristics	Yes	49	38.0
		No	80	62.0
	Lack of knowledge	Yes	99	76.7
		No	30	23.3
	Parent’s own disability	Yes	11	8.5
		No	118	91.5
	Issues in the home environment because of elderly care	Yes	0	0.0
		No	129	100.0
	Lack of support for family environment	Yes	7	5.4
		No	122	94.6
	Child withdrawal	Yes	10	7.8
		No	119	92.2
Negative	Failure to recognize characteristics	Yes	23	74.2
		No	8	25.8
	Lack of knowledge	Yes	20	64.5
		No	11	35.5
	Parent’s own disability	Yes	9	29.0
		No	22	71.0
	Issues in the home environment because of elderly care	Yes	0	0.0
		No	31	100.0
	Lack of support for family environment	Yes	2	6.5
		No	29	93.5
	Child withdrawal	Yes	0	0.0
		No	31	100.0
Optimistic	Failure to recognize characteristics	Yes	19	73.1
		No	7	26.9
	Lack of knowledge	Yes	21	80.8
		No	5	19.2
	Parent’s own disability	Yes	3	11.5
		No	23	88.5
	Issues in the home environment because of elderly care	Yes	0	0.0
		No	26	100.0
	Lack of support for family environment	Yes	0	0.0
		No	26	100.0
	Child withdrawal	Yes	0	0.0
		No	26	100.0
Indifferent	Failure to recognize characteristics	Yes	10	52.6
		No	9	47.4
	Lack of knowledge	Yes	14	73.7
		No	5	26.3
	Parent’s own disability	Yes	6	31.6
		No	13	68.4
	Issues in the home environment because of elderly care	Yes	2	10.5
		No	17	89.5
	Lack of support for family environment	Yes	2	10.5
		No	17	89.5
	Child withdrawal	Yes	0	0.0
		No	19	100.0
None	Failure to recognize characteristics	Yes	0	0.0
		No	17	100.0
	Lack of knowledge	Yes	1	0.5
		No	16	94.1
	Parent’s own disability	Yes	0	0.0
		No	17	100.0
	Issues in the home environment because of elderly care	Yes	0	0.0
		No	17	100.0
	Lack of support for family environment	Yes	0	0.0
		No	17	100.0
	Child withdrawal	Yes	0	0.0
		No	17	100.0

Case study

When providing support for the child’s difficulties, cases were chosen that took into account both the parent’s characteristics and difficulties. These two cases were chosen for several reasons. First, diligent parents represented the largest group among parental characteristics in our dataset. Unlike other parental types, they rarely overlapped with multiple parental difficulties, which reduced the likelihood of confounding factors when analyzing the interaction between parental characteristics and child difficulties. Second, withdrawal was chosen as the child's difficulty because it was both clinically recognizable and consistently documented in support records, which allowed for the tracking of observable changes. Finally, these two cases had sufficiently detailed longitudinal records, enabling a retrospective qualitative analysis of the intervention process within the ICF framework. Together, these factors ensured that case selection was based on representativeness and analytic feasibility rather than arbitrariness. The support’s effectiveness was analyzed qualitatively, using activities and participation within the framework of the ICF, thereby analyzing the relationship between the child’s difficulties (personal factors) and the parent’s characteristics and difficulties (environmental factors).

Case A: A Case of Gradual Support for a Child With ASD and Anxious Parents, Leading From Home Visits to Center-Based Support

This case was of a boy who was nine years and nine months old at the start of the program with a diagnosis of ASD, ending when he was 14 years and one month old. He experienced strong anxiety, making it difficult to attend school. Additionally, his parents found it difficult to cope with the child’s behaviors or characteristics associated with the diagnosis of ASD. His identified difficulties included "poor social communication," challenges in "interpersonal relationships," and "refusal to be involved with people outside of the family." The parents’ difficulties included "child withdrawal" and "failure to recognize his characteristics." At the beginning of the intervention, the child’s anxiety was severe, and an OT provided individual support at home. The child gradually accepted the OT’s support and became able to receive assistance at our center. Throughout the intervention, the setting was gradually changed, and activities that the parent and child could work on together were suggested, increasing opportunities for them to share experiences. This suggests that the parents may have gradually come to accept the characteristics of their child (see Appendix A).

Case B: Intervention Case Aiming to Improve Functions and Prepare for School Enrollment for a Child With ASD and Sensory Sensitivities

The child in his case was a boy in his final year of kindergarten, with an IQ of 79 and a diagnosis of ASD. The case presented sensory sensitivities, a feature of ASD, alongside the ongoing development of the ability to perform tool manipulation. The children’s difficulties identified were "refusal to get involved outside of the family." The parents’ difficulties included "child withdrawal" as well as "lack of knowledge." The OT progressively presented activities that the child could readily accept, with the aim of promoting the child’s physical functions. This process enabled the child to acquire the skills necessary for activities in elementary school. Further, in response to the environmental change in school enrollment, the intervention proceeded while information was gathered from the parents, and an environment was created where the child could actively participate (see Appendix B).

In both of these cases, the focus was on the child’s mental functions, such as anxiety, and physical functions, such as upper limb function. The characteristics of both the child and the parents were assessed, and appropriate activity suggestions and environmental adjustments were made. This intervention brought about changes in the child’s activities and participation in the ICF framework, producing positive impacts for both the child and the parents.

With respect to parental difficulties, which play the role of an environmental factor, it was suggested that adjusting the approach to the parents following assessment could be effective. In particular, for parents who fail to recognize the characteristics of their child’s challenges, a positive change in attitude, such as asserting that they wished to do what they could for the child, was observed as the parents experienced the positive changes in the child. Thus, support that focuses on the changes that take place in the parents’ mental and physical well-being alongside the child’s progress may have led to the child, then in a state of withdrawal, to become able to interact with others through going to the center. In particular, the incorporation of parental involvement as part of the support is suggested to promote understanding and acceptance of the child’s characteristics.

However, for parents who lack knowledge, explanations of the characteristics and developmental stages of ASD were provided, and they were encouraged to share information on the child’s behavior at home and school; this may have deepened their understanding of the child. It can be inferred that this engagement with the parents was a factor in enhancing the effectiveness of the support. In addition, these results suggest that the presence of an OT in a child development support facility plays a role in connecting the families (parents) of children refusing to attend school with the schools (teachers).

## Discussion

This study examined support records from child development support and after-school day services led by rehabilitation professionals, including OTs and PTs, focusing on interventions that were tailored to the characteristics of children and their parents.

The results revealed six life challenges extracted from the records of 222 service users. Regarding children’s difficulties, interpersonal relationships were the most frequently reported challenge, but this often overlapped with other difficulties. In particular, poor social communication and self-injurious behavior were frequently seen with interpersonal relationship challenges and restlessness or strong tantrums (see Figure 1). Furthermore, self-injurious behavior overlapped with all other difficulties, suggesting that such behavior is a manifestation of a combination of complex factors, as seen in its association with impairments in adaptive functioning, communication skills, IQ, sleep, atypical sensory processing, and impulsivity/hyperactivity [11]. Despite the exclusion of depressive symptoms from this study's assessment, future research should investigate the potential link between self-injurious behavior and depression in children to enhance our understanding and inform more comprehensive intervention strategies.

Of the participants, 146 had diagnoses of ASD, ADHD, and/or intellectual disability. These produced characteristics such as sensory hypersensitivity or hyposensitivity, anxiety, and disrupted sleep-wake cycles, which were factors that required support from OTs and/or PTs. In the provision of support, sensory and motor assessments were performed to create individual support plans. This aligns with reports indicating that the integration of physical and sensory evaluations in school settings can support ASD children’s overall well-being [12]. In addition, the case reports indicated that OTs also suggested the involvement with gross motor play. This is consistent with findings showing that more frequent engagement in physical activities is associated with higher levels of adaptive functioning [13]. Such findings indicate that CDSMs who are OTs or PTs developed support to improve children’s health, daily living, sensory and motor skills, cognition and behavior, language and communication, and interpersonal relationships and social skills, in relation to the children’s characteristics. The service users’ support records also provided insight into the characteristics of the children’s parents. The interviews conducted by OTs and PTs with the parents identified four types of parental characteristics.

The most frequent characteristic among parents was diligence. A survey of children’s living conditions conducted by the Yamagata Prefecture suggests a high level of awareness among parents regarding education [14]. The results of this study indicate that diligent parents exhibited different tendencies in response to difficulties relative to parents who were characterized as optimistic or negative. In particular, diligent parents were less likely to show an overlap between lack of knowledge regarding involvement with a child with disabilities and unwillingness to acknowledge the child’s disability. This suggests that, while diligent parents recognized their child’s disability, they may not have sufficient access to the necessary information. This background implies a regional scarcity of facilities that are capable of providing support that is tailored to children’s specific needs, indicating parents’ need for such child development support services.

The next most frequent characteristic was negative. Over 80% of negative parents were parents of children with diagnoses. Mothers tend to be overinvolved in their children’s developmental disabilities [15]. In addition, parents of children with disabilities are more likely to use maladaptive strategies of emotion regulation and are less likely to use adaptive strategies than typically developing children [16]. Further, the degree of depression seen in parents of children with autism and intellectual disabilities is high, where mothers are more prone to depression than fathers [17,18]. Psychological flexibility in the parents of children with disabilities is associated with the positive aspects of raising a child with a disability [19]. Peer support interventions produce improvements in the well-being and quality of life of parents and caregivers, in support of current practices [19]. To enhance the quality of life of families, it is crucial to begin to manage the characteristics of ASD immediately following diagnosis and to implement psychoeducation and support programs for parents [20]. Collaboration between parents and OTs requires an individualized approach, and therapists must adopt a facilitative mindset when engaging with parents [21]. Further, a shift in practice from therapist-led, child-centered care to family-centered care should be promoted [22]. In addition, promoting parents’ autonomous decision-making by parents is essential for adolescents who have disabilities and forms the basis for the maturation of their self-determination [23]. Moreover, the two case studies demonstrate that OTs assess the child’s physical and mental functions and utilize the child’s preferred play (activities) to gradually change the environment and adjust stimuli during the intervention. They also simultaneously acknowledged the parents' difficulties and provided interventions that considered these influences as environmental factors affecting the child's activities, participation, and psychological functioning.

Thus, to address the life challenges of service users, it is necessary to understand the characteristics of both parents and to clarify the challenges that are faced by the family. In this study, in some instances, the parent-child relationship became strained as a result of diligent parents’ lacking knowledge concerning their child’s disability characteristics. This indicates that child development support should incorporate family support in addition to supporting the child. OTs and PTs in Japan are generally aware of this, but have not been able to provide sufficient support. Further, the institutional positioning of OTs and PTs in child development support and after-school day services is not mandatory. For this reason, there are few OTs and PTs who are engaged in the developmental field and in clinical practice, and the paucity of opportunities for on-the-job training in practical settings can be considered a challenge.

Limitations

This study has several limitations stemming from the data source and the methodology employed.

Methodological Limitations

Our approach utilized a systematic case classification method performed by OTs. This was necessary because no currently validated Japanese instrument exists to capture the nuanced attitudes and behaviors of parents within child development support services. While the classification was grounded in clinical reasoning and conducted collaboratively within a multidisciplinary team, we acknowledge that this introduces potential evaluator bias. We believe, however, that the multidisciplinary nature of the coding reduced the risk of arbitrary classification.

Data and Scope Limitations

The analysis was exclusively based on data extracted from the OTs from the records. Consequently, the findings are limited to the OT perspective, preventing us from identifying potential differences that might arise if records from other professions, such as nursery teachers, were analyzed. This limitation also meant we were unable to clearly delineate the specific expertise of the OTs in this context. Furthermore, this study was unable to conduct a comprehensive investigation into the support content of child development and after-school day services across Yamagata Prefecture. Future research aimed at understanding the regional current state requires analyzing existing data on facility numbers and utilization, in addition to conducting questionnaire surveys and interviews with facility representatives to grasp the specific details of their support.

## Conclusions

This study analyzed support records from child development support and after-school day services led by OTs, focusing on interventions that were tailored to the characteristics of children and their parents. An analysis revealed that the main challenges for children were interpersonal relationship problems and restlessness, and tantrums, while parents struggled with a lack of knowledge. These difficulties often overlapped, with children exhibiting self-injurious behavior showing signs of all other challenges. The most common group of parents, characterized as "diligent," tended to recognize their child's characteristics but lacked the necessary information. OT interventions, which considered the characteristics of both the child and the parent, were found to be effective through the use of play and environmental adjustments. From these results, it is suggested that to resolve the life challenges of service users, it is necessary to clarify the challenges that are faced by families, taking the characteristics of both parents into account. Child development support is not only crucial for service users but also for family support. However, the current shortage of OT and PT personnel is a serious issue, which makes it challenging to establish and develop support systems. Future research should address these challenges and proceed toward building a comprehensive support system with multidisciplinary collaboration.

## Appendices

Appendix A 

**Table 5:** Case A: a case study of an intervention that led from home visitation to daycare support through step-by-step support for an anxious ASD child and his parents

First period	June to July 2019 (fourth grade) *Relationship: orienting
Case characteristics	This case was a boy in the fourth grade who was diagnosed with autism spectrum disorder (ASD). He was being treated with aripiprazole and ramelteon. His anxiety was intense, especially in crowded places, such as school. He had difficulty communicating with people of his own age. He was detail-oriented and obsessive. He was always nervous and was sensitive to minor stimuli including having difficulty with loud noises. He did not interact with anyone outside his family, and he spent most of his time watching YouTube on his mother’s phone.
Intervention goal	To establish a bilateral relationship with his assigned occupational therapist (OT).
Intervention	OT visited the boy at his home, an environment in which he felt safe, and the meeting went at his pace. Specific activities included watching his favorite YouTube videos with him and communicating with him regarding his favorite things, such as the sound of trains, so that he could feel the joy of communicating his feelings to others.
Case observations	At the first visit, he was very anxious, and OT could not interact with him directly; however, OT later came to the room where OT was by himself to greet him. OT tried to make him feel at ease through listening to his story and sympathizing with him. Little by little, his anxiety toward the OT appeared to dissipate; at the second visit, he came down a short time after the OT’s arrival. From the third visit on, he began to wait for the OT in the living room on the first floor.
Family’s intervention and relationship	Before the intervention, the mother wore a hat pulled low over her face and looked downcast and concerned. The mother’s statements, “If he doesn’t like it, I want you to pull back,” and “If you are too loud, he might get scared.” She indicated her fear that her son would be hurt. The mother appeared diligent, but also anxious, introverted, and sensitive.
Second period	August to December 2019 (fourth grade) Relationship: orientation-identification
Case characteristics	The anxiety was at a lower level but it was still strong. Tension was less than at the beginning of the intervention; in early August 2019, the boy’s main activity was watching YouTube at home, but over the course of the previous few months, the range of interests and activities has broadened.
Intervention goal	To expand the place of activity outside the home.
Intervention	Enjoying activities with the OT will increase the boy’s sense of self-efficacy. Increased self-efficacy will foster the desire to spend time with people other than the person in charge and to engage in activities outside the home.
Case observations	At the beginning of August 2019, the boys mainly watched YouTube, but at this later observation, he was able to play with his friends at home. The involvement of the OT may have also caused him to feel pleasure in talking to someone outside of his family; in September, he was able to go to the train station and shopping malls; in October, he was able to go to school at the time of the parent-teacher conference. His parents provided him with a video game console for his birthday in mid-October, which provided him with a wider range of activities, beyond watching YouTube on his mother’s phone.
Family’s intervention and relationship	During the early stages of the intervention, the boy’s parents were concerned and felt that they did not know what to do. However, as more than three months passed since the intervention and positive changes were seen, they showed an increasing desire to do what they could.
Third period	January to June 2020 (fourth to fifth grade) Relationship: identical
Case characteristics	The boy’s anxiety was moderate. He no longer had high nervousness when in a safe environment or with a safe partner. The range of interests and activities expanded. However, in unfamiliar environments, tension was high and it was still difficult for him to continue going to school.
Intervention goal	To spend time with the OT outside his home.
Intervention	To expand the boy’s activities outside of his home, OT played badminton with him in front of his home. Due to the awkwardness of the gross motor aspect, he sometimes did not well. However, by providing advice tailored to his physical functions, he gradually improved, leading to an increased sense of self-efficacy. Furthermore, as the OT continued to work with him, he became confident that he would be able to improve if he kept at it without giving up. The fact that the activity was conducted by three people (the person in question, his/her father, and the person in charge) allowed the participants to feel the fun of doing activities with more than three people. We also created an environment in which the boy could communicate what he liked outside of the house through playing games and watching YouTube at the front door.
Case observations	In May 2020, he was able to go to the day service, followed by a visit to the dentist where he wore earphones and a short visit to a friend’s birthday party. One day, he made an effort to go to the dentist, and then went to see his favorite steel tower and was able to play his favorite game during the day service. On that day, he said, “This is the best day ever!” The OT’s intervention appears to have increased the boy’s confidence, led to a wider range of outdoor activities, and allowed him to face more challenges and obtain more enjoyment. However, it was still difficult for him to continue going to school.
Family’s intervention and relationships	The mother appeared diligent and concerned, but she was becoming more open to new methods, such as those outside the home. The mother herself was willing to try new experiences as her son grew older.
Fourth period	June to December 2020 (fifth grade) Relationship: identification
Case characteristics	Anxiety was still moderate. The boy’s activities are expanding beyond the borders of the prefecture, and his tolerance for places and people has expanded. However, he still had difficulty continuing to attend both school and day-care.
Intervention goal	To expand the place of activities and increase the number of people that he can spend time together with.
Intervention	Where a staff member was present along with the OT in charge, the staff member was recognized as a person with whom the boy could feel at ease. The places of activities were expanded to outside the prefecture (Niigata and Sendai) in the third period. OT was conscious of increasing the boy’s sense of self-efficacy, telling him, “It’s amazing that you know something that (the staff member in charge) doesn’t know.”
Case observations	In August and September 2020, the range of the boy’s activities expanded to the point where he could travel out of the prefecture with his family to see his favorite pylons. This confidence in his ability to have fun outside of their own home led them to expand their daily activities, such as walking around the neighborhood and shopping together. In December 2020, he was able to participate in activities with staff members other than the one in charge of the day service. While the frequency of his visits to the day service increased, it did not continue, and he still had difficulty attending school. Anxiety, including about the weather, persisted, to the point of vomiting.
Family intervention and relationship	The OT’s home visits led to visits to the home by the consultation support specialist and school teachers, improving the receptivity of the parents. It was clear that the parents were became more willing to be there for the individual. At the same time, they were willing to fulfill his request without sparing time or money, even for outings to distant places.
Fifth period	January 2021 to March 2022 (fifth grade to elementary school graduation) Relationship: identification-pioneering use
Case characteristics	While the boy’s anxiety was moderate, he was beginning to be able to express his anxieties and requests to others. In addition to his YouTube activities, he also expanded his hobbies, such as going to see pylons, taking pictures, and researching and buying hand spinners on the Internet.
Intervention goal	Continue going to the daycare. To increase the number of people who were working together.
Intervention	It was difficult for the client to maintain a stable commute, so a safe environment was created. His habit of walking began after he began going to the daycare, and decreased activity during the winter became less. The trauma of riding a bicycle was overcome, and the residents were encouraged to increase their sense of self-efficacy. Visits were reduced due to the increase in commuting but continued in response to requests from the client. The fact that he could express his feelings was a significant development. We intervened to make the expression of feelings a positive experience for him. When the patient was anxious, consultation with a medical institution and medication adjustment were implemented.
Case observations	From January to June 2021, there were periods of continuous attendance but also periods of inability to attend. Anxiety increased for a time, but then, it stabilized. From July to December 2021, the client could continue going to the daycare. From October onward, the intervention of another female staff member was accepted. However, it was difficult to expand the scope of the boy’s interactions to others beyond the person in charge and the female staff member. From January to March 2022, events were held for elementary school graduation and middle school enrollment. The boy appeared to be feeling positive about entering junior high school and was able to attend a farewell party for his support class. For the first time in several years, he was able to spend an hour or so at school in an environment with my child.
Family’s intervention and relationship	The mother spoke more with the OT about her concerns about his charge. The relationship between the parents and the boy was stable. The parents came to the daycare together more often, and the father talked with the school counselor and cooperated with him more often.
Sixth period:	April 2022 to April 2024 (middle school enrollment to 2024) Relationship: pioneering use
Case characteristics	The boy’s anxiety is moderate. His nervousness increases in environments having many children of the same age, but he is at ease in other situations. The range of his activities has expanded as the number of people with whom he can be involved in activities has increased.
Intervention goal	Increase the number of people who can spend time together with peace of mind. To become independent in ADLs.
Intervention	Gradually, the number of staff who were involved in the day-care center increased. In addition, both the boy and his family began to look toward the future, beginning to ask for advice on how he could become more independent. We encouraged the boy to gradually do by himself what his family used to do with him or for him.
Case observations	After he entered junior high school, the boy’s condition stabilized. Continued attendance became possible, and, as a result, he was able to relate to almost all of the staff members. He was also able to continue weekly after-school visits to the school, perhaps because he was a good match with his homeroom teacher during his first year of junior high school. He was also able to accept a change in his homeroom teacher. However, he was not able to attend school at times when other students were present.
Family’s intervention and relationship	His relationship with his parents was stable; although age-appropriate rebelliousness was observed, it did not become a major problem. Before he entered junior high school, his parents were able to consult other people besides the school counselor and social services.
*Stages based on Hildegard E. Peplow’s Nursing Theory of the Nurse-Patient Interpersonal Relationship.	

Appendix B 

**Table 6:** Case B: case study of intervention to improve upper limb function for school readiness in an ASD child with sensory hypersensitivity ASD: autism spectrum disorder; OT: occupational therapist

First period	November 2022 to March 2023
Case characteristics	This case was an older boy with an IQ was 76 (Tanaka-Binet Intelligence Scale), diagnosed with ASD. He was a picky eater. He had poor hand pressure and could not hold a pencil. He had a resistance to getting wet. At preschool, he could not go to the toilet and was afraid to sit on the toilet seat at home. He could not try things that he had never tried, threw tantrums when things did not go well, did not like to do the same things as everyone else, and easily got lost in his own world.
Intervention goal	Becoming part of a day-care environment.
Intervention	The boy’s favorite activities were assessed; he was asked to choose his favorite activity from two options. The OT created a schedule and provided perspective. The OT incorporated communication practice when borrowing favorite toys. Gross and fine motor activities were implemented.
Case observations	He felt comfortable attending the daycare. He rejected few of the activities we proposed, and we performed gross and fine motor activities while incorporating his favorite activities. We incorporated writing activities and observed that his writing pressure increased about two months after the intervention.
Family’s intervention and relationship	The mother appeared cooperative about the rehabilitation, but the grandparents seemed to have difficulty accepting it.
Second period	April 2023 to March 2024 (first grade)
Case characteristics	In May 2023, the boy wrote more characters and drew more pictures than before. However, he appears to struggle with hiragana having separate parts such as は and か and complex hiragana such as ぬ and ね (these are often left blank in homework assignments). In numbers, 4 is a mirror character, but other numbers can be copied.
Intervention goal	To become accustomed to a new environment after elementary school. To concentrate in listening to a conversation. To be able to express his feelings to others.
Intervention	The boy was also able to challenge himself with respect to writing assignments, and gradually, his writing became more legible. Because it was easier for him to concentrate in a tranquil environment, OT chose such a quiet space for his activities. Even when he was reluctant to go to school, he was able to go to the daycare easily. The mother informed OT that “he seems to enjoy being physically active.” Therefore, physical activity was also continued.
Case observations	When he first entered elementary school, the boy showed no signs of refusal to go to school. During his time at the daycare, he was very motivated to try his hand at scissors, which he felt he was not good at. In June, he was able to separate himself from his mother at the daycare. His mother reported that he was absent from school from November 28 to December 1, 2023. He complained that he felt “heavy." Despite his usual goal of doing things right, his mother thought that the teacher in his support class could have been the reason for his absence, saying, “Let’s do it again, even if it’s wrong.” Something happened at school, he cried, and he began to say that he was afraid of school. In February, he went to the school twice a week to get his prints and timetable. He said that he was afraid of the ogres, and in March, he was anxious that he might not be able to go back home because of the snow.
Family’s intervention and relationship	The father accompanied the boy to the daycare. The mother thought that the child had been “pushing himself too hard” in response to the above-mentioned frequent absences from school. While the boy was not going to school, he was watching YouTube and playing with Lego blocks by himself. The mother felt concerned that he would become a recluse.
Third period	April to December 2024 (second grade)
Case characteristics	The boy’s writing pressure is stronger than before, but left–right recognition is difficult.
Intervention goal	To gain experience of playing well with friends and learning rules and social skills. With the help of others around them, they gain experience in compromise and negotiation and increase confidence in what they can do.
Intervention	The activities that are proposed revolve around movement. The boy has been able to work on some of the activities without any major rejection of any. In addition, the acceptance is not bad when another person in charge of the project is opposed.
Case observations	In the spring of 2024, the boy appeared a little stiff in the daycare. In June 2024, he was prescribed Melatobel. He was able to attend school during the midterm break and study without a chaperone in the third period. He could attend daycare without breaks and did not refuse participation in activities. He is able to keep trying even when he is tired, unlike in the past.
Family’s intervention and relationship	Because the homeroom teacher went on maternity leave in July, my mother was concerned about whether she could maintain the current pace. However, the substitute teacher who arrived after the summer break had a similar approach to the previous teacher. Therefore, the mother felt relieved, as she was able to relate to the teacher.

## References

[1] (2024). Japanese Law Translation. Child Welfare Act. https://www.japaneselawtranslation.go.jp/ja/laws/view/4035.

[2] (2024). Current status of child development support and after-school day care services, etc. [PDF in Japanese]. https://www.mhlw.go.jp/content/12401000/001023067.pdf.

[3] (2024). Guidelines for after-school day services [PDF in Japanese]. https://www.mhlw.go.jp/file/05-Shingikai-12201000-Shakaiengokyokushougaihokenfukushibu-Kikakuka/0000082829.pdf.

[4] (2024). Child Development Support Services “when child development support is provided outside of child development support centers” Outline [PDF in Japanese]. https://www.mhlw.go.jp/seisakunitsuite/bunya/hukushi_kaigo/shougaishahukushi/kaiseihou/dl/sankou_111117_01-07.pdf.

[5] (2024). Child development support guidelines [PDF in Japanese]. https://www.mhlw.go.jp/file/06-Seisakujouhou-12200000-Shakaiengokyokushougaihokenfukushibu/0000171670.pdf.

[6] (2024). Survey of workers at day-care center support facilities for handicapped children [PDF in Japanese]. https://www.mhlw.go.jp/file/06-Seisakujouhou-12200000-Shakaiengokyokushougaihokenfukushibu/0000178193.pdf.

[7] (2024). Current status of day-care support for children with disabilities, etc. “Material on p. 11 added to Document 3 of the first meeting for consideration” [PDF in Japanese]. https://www.mhlw.go.jp/content/12401000/000801033.pdf.

[8] (2024). International Classification of Functioning, Disability and Health (ICF). https://www.who.int/standards/classifications/international-classification-of-functioning-disability-and-health.

[9] Amonkar N, Su WC, Bhat AN, Srinivasan SM (2021). Effects of creative movement therapies on social communication, behavioral-affective, sensorimotor, cognitive, and functional participation skills of individuals with autism spectrum disorder: a systematic review. Front Psychiatry.

[10] Su WC, Srinivasan S, Bhat AN (2025). Effects of creative movement, general movement, or seated play interventions on motor performance in children with autism spectrum disorder: a pilot randomized controlled trial. Res Autism Spectr Disord.

[11] Vandewalle K, Melia Y (2021). Psychosocial and behavioural factors associated with self injurious behaviour (SIB) in individuals with autism spectrum disorders (ASD). Res Autism Spectr Disord.

[12] Trudel SM, Winter EL, Fitzmaurice B, Norman G, Bray CR (2022). Integration of physical health and sensory processing assessment for children with autism spectrum disorder in schools. Psychol Sch.

[13] Neville RD, Draper CE, Cooper TJ, Abdullah MM, Lakes KD (2021). Association between engagement in physical activity and adaptive behavior in young children with autism spectrum disorder. Ment Health Phys Act.

[14] (2025). Yamagata prefecture children's living conditions survey. http://https://www.pref.yamagata.jp/010002/kenfuku/kosodate/shien/kodomonoseikatsujittaichousa.html.

[15] Acar S, Chen CI, Xie H (2021). Parental involvement in developmental disabilities across three cultures: a systematic review. Res Dev Disabil.

[16] Keleynikov M, Benatov J, Cohen N (2023). Emotion regulation among parents raising a child with disability: a systematic review and conceptual model. J Child Fam Stud.

[17] Millaku J, Kraja-Bardhi E (2023). Depression among parents of disabled children. Int J Innov Res Sci Stud.

[18] Dimachkie Nunnally A, Factor RS, Sturm A (2023). Examining indicators of psychosocial risk and resilience in parents of autistic children. Front Behav Neurosci.

[19] Gur A, Reich A (2023). Psychological flexibility of parents of children with disabilities: a systematic literature review. Res Dev Disabil.

[20] Lancaster K, Bhopti A, Kern ML, Taylor R, Janson A, Harding K (2023). Effectiveness of peer support programmes for improving well-being and quality of life in parents/carers of children with disability or chronic illness: a systematic review. Child Care Health Dev.

[21] Papadopoulos A, Siafaka V, Tsapara A, Tafiadis D, Kotsis K, Skapinakis P, Tzoufi M (2023). Measuring parental stress, illness perceptions, coping and quality of life in families of children newly diagnosed with autism spectrum disorder. BJPsych Open.

[22] Klatte IS, Ketelaar M, de Groot A, Bloemen M, Gerrits E (2024). Collaboration: how does it work according to therapists and parents of young children? A systematic review. Child Care Health Dev.

[23] Taub T, Werner S (2023). Perspectives of adolescents with disabilities and their parents regarding autonomous decision-making and self-determination. Res Dev Disabil.

